# The *Acinetobacter baumannii* model can explain the role of small non-coding RNAs as potential mediators of host-pathogen interactions

**DOI:** 10.3389/fmolb.2022.1088783

**Published:** 2022-12-21

**Authors:** Meysam Sarshar, Daniela Scribano, Anna Teresa Palamara, Cecilia Ambrosi, Andrea Masotti

**Affiliations:** ^1^ Research Laboratories, Bambino Gesù Children’s Hospital, IRCCS, Rome, Italy; ^2^ Department of Public Health and Infectious Diseases, Sapienza University of Rome, Rome, Italy; ^3^ Laboratory Affiliated to Institute Pasteur Italia-Cenci Bolognetti Foundation, Department of Public Health and Infectious Diseases, Sapienza University of Rome, Rome, Italy; ^4^ Department of Infectious Diseases, National Institute of Health, Rome, Italy; ^5^ Department of Human Sciences and Promotion of the Quality of Life, San Raffaele Roma Open University, Rome, Italy; ^6^ IRCCS San Raffaele Roma, Rome, Italy

**Keywords:** *Acinetobacter baumannii*, small RNAs (sRNAs), host-pathogen interactions, non-coding RNAs, antibiotic-resistance, outer membrane vesicles (OMVs)

## Abstract

Bacterial small RNAs (sRNAs) research has accelerated over the past decade, boosted by advances in RNA-seq technologies and methodologies for capturing both protein–RNA and RNA–RNA interactions. The emerging picture is that these regulatory sRNAs play important roles in controlling complex physiological processes and are required to survive the antimicrobial challenge. In recent years, the RNA content of OMVs/EVs has also gained increasing attention, particularly in the context of infection. Secreted RNAs from several bacterial pathogens have been characterized but the exact mechanisms promoting pathogenicity remain elusive. In this review, we briefly discuss how secreted sRNAs interact with targets in infected cells, thus representing a novel perspective of host cell manipulation during bacterial infection. During the last decade, *Acinetobacter baumannii* became clinically relevant emerging pathogens responsible for nosocomial and community-acquired infections. Therefore, we also summarize recent findings of regulation by sRNAs in *A. baumannii* and discuss how this emerging bacterium utilizes many of these sRNAs to adapt to its niche and become successful human pathogen.

## Introduction

Both coding and non-coding RNA molecules (ncRNAs) have attracted great interest for their ubiquitary roles in almost all cellular and evolutionary processes of living organisms (Wu et al., 2021; Yates et al., 2021). By definition, both coding and ncRNAs are transcribed from DNA but ncRNAs are not translated into functional proteins (Han and Chen, 2015; Diamantopoulos et al., 2018). Remarkably, ncRNAs are expressed at specific stages of cell’s development, and involved in gene modulation in response to external stimuli (Szymański et al., 2003; Li et al., 2021). Long (>200 nt) and short (<200 nt) ncRNAs were found in both eukaryotes and prokaryotes (de la Fuente et al., 2012; Deogharia & Gurha, 2022).

Discovered as early as the 1950s, ncRNAs were initially deemed as by-products of huge transcripts with negligible biological roles (i.e., “junk” RNAs). Thereafter, the development of advanced technologies revolutionized the exploration of ncRNAs, resulting in the discovery of distinctive ncRNA species, both cellular and circulating, with different biological roles in many human diseases (Cech & Steitz, 2014; Felli et al., 2017; Hung et al., 2018; Slack & Chinnaiyan, 2019; Tait et al., 2020; Toden et al., 2021). Among all, the best-studied family of ncRNAs is miRNAs that able to interact with other ncRNAs as well as various types of mRNA transcripts in a regulatory crosstalk known as competing endogenous RNAs (ceRNAs) leading to an additional post-transcriptional levels (Agirre & Eyras, 2011; Ala, 2020; Li et al., 2020).

Recently, Seal and colleagues have provided a guide to the nomenclature of human ncRNAs genes by reviewing each major class based on the HUGO Gene Nomenclature Committee (HGNC; http://www.genenames.org), providing an updated database and useful resources available online (Da Sacco et al., 2011; Seal et al., 2020). They function as post-transcriptional regulators of their mRNA targets resulting in the translational repression and/or degradation (Djebali et al., 2012; Catalanotto et al., 2016; Hung et al., 2018; Sun et al., 2018). Moreover, different kinds of ncRNAs can interact with each other and/or with DNA, as well as other RNA classes and proteins (Baldassarre & Masotti, 2012; RamónCajal et al., 2019; Sun et al., 2020). Various ncRNAs have been discovered to be encapsulated and transported through exosomes, microvesicles, and apoptotic bodies, all referred to as extracellular vesicles (EVs), ranging from ∼30 to 150 nm in diameter (Fischer & Deindl, 2021; Li et al., 2021). Exosomal ncRNAs, mainly miRNAs, lncRNAs, and circRNAs, have been reported to be expressed by different cells in several physiological and pathological conditions (Li et al., 2021). Once secreted, they are transported by body fluids, such as serum, plasma, urine, saliva, etc, to recipient cells where they could influence different functionalities (Li et al., 2021; Qiu et al., 2021; Wang et al., 2022) or can be employed as useful diagnostic biomarkers of paediatric diseases (Masotti et al., 2017a; Masotti et al., 2017b; Paolini et al., 2022a; Paolini et al., 2022b; Felli et al., 2022).

### Insights into bacterial sRNAs

Further investigations have led to explore bacterial RNA-dependent mechanisms associated with ncRNAs. In bacteria, a class of small ncRNAs (i.e., sRNAs), are gene expression mediators at both transcriptional and post-transcriptional levels. So far, hundreds of sRNA molecules ranging from approximately 50–400 nt were found ubiquitously in different bacterial species, among which sRNAs from *Escherichia coli* (*E. coli*) are the most studied (Storz et al., 2011; Kang et al., 2013; Pérez-Reytor et al., 2017; Mediati et al., 2021). Using next-generation sequencing (NGS), Lee and Hong identified several short sRNAs (ca. 26 nt) in *Streptococcus mutans*, and proposed the term “microRNA-size,” small RNA (msRNA) (Lee & Hong, 2012). Furthermore, very small RNAs (vsRNAs <16 nt) as well as tRNA-derived fragments (tRFs), belonging to the category of vsRNAs, were recently discovered in bacteria (Diallo et al., 2022). Interestingly, tRFs seem to represent the majority of sncRNA types in bacteria as well as in eukaryotes (Plante et al., 2012; Lambert et al., 2021) suggesting a conserved evolutionary strategy to modulate gene expression from bacteria to eukaryotes.

Nowadays, thanks to the development of RNA sequencing, it is known that production and regulation of bacterial sRNAs is coordinated through other components, including others sRNAs, mRNAs, and remarkably sRNA-binding proteins (sRBPs) (Adams & Storz, 2020; Goldberger et al., 2021). Several sRBPs (e.g., Hfq, ProQ, and CsrA) are involved in facilitating sRNA-mRNA base-pairing in *Enterobacteriaceae*, mainly investigated in *E. coli* and *Salmonella enterica* serovar Typhimurium (Storz et al., 2011; Kim & Kwon, 2013; Klein & Raina, 2017; Goldberger et al., 2021). In *E. coli*, the Hfq protein, an RNA chaperone, enhances base pairing between a specific sRNA and its mRNA target (Waters & Storz, 2009; De Lay et al., 2013; Vazquez-Anderson & Contreras, 2013). Hfq interacts with at least 40% of the known sRNAs and has the ability to interact not only with other ncRNAs, but also with longer RNAs (i.e., rRNAs and tRNAs) and most importantly with many different mRNAs (Pichon & Felden, 2007; Bandyra et al., 2012; Waters et al., 2017; Quendera et al., 2020). The functional importance of sRBPs in “sRNA-mediated” regulation of gene expression in bacteria was summarized previously (Pichon & Felden, 2007).

### Host-pathogen interactions mediated by sRNAs

sRNAs secreted by bacteria may play important roles not only in microbe–microbe but also in host–microbe interactions (Ahmadi Badi et al., 2020). In both Gram-negative and Gram-positive bacteria, two major characteristics of sRNAs have been identified, the cis- and trans-encoded sRNAs. These sRNAs, once coupled to their RNA target(s) lead to inhibition or activation of target gene expression (e.g., genes responsible for controlling bacterial adaptation to environmental changes and virulence) through a variety of post-transcriptional gene regulation mechanisms (Li et al., 2012; Felden & Gilot, 2018; Chakravarty & Massé, 2019; Felden & Augagneur, 2021; Millar & Raghavan, 2021). Usually, cis-encoded sRNAs are transcribed from the same genetic locus but in the opposite direction to their single RNA target against which they pair, whereas trans-encoded sRNAs are transcribed in a distant locus (respect to theis targets) and exert their regulatory functions by partially pairing to their RNA targets (Adams & Storz, 2020; Carrier et al., 2020; Dell’Annunziata et al., 2020; Jørgensen et al., 2020). Several bacterial sRNAs are considered as master regulators in that they are able to modulate the expression of transcriptional factors responsible for the activation of virulence genes or they can target the quorum-sensing regulatory components by regulating multiple mRNA targets (Quorum Regulatory RNAs, QRR) (Broach et al., 2012; Pérez-Reytor et al., 2017; Felden & Cattoir, 2018; Janssen et al., 2020; Lalaouna et al., 2021). Hence, sRNAs are emerging as key controllers of central regulatory circuits determining the bacterial lifestyle. sRNAs encoded on pathogenicity islands (PAIs) or virulence plasmids are prime candidates to directly control the expression of virulence genes (Papenfort & Vogel, 2010; Papenfort & Vogel, 2014). Several types of RNA-based regulatory mechanisms as well as their interactome in pathogenic bacteria have been reported so far (Sridhar & Gunasekaran, 2013; Waters et al., 2017; Chakravarty & Massé, 2019). The most intriguing types of bacterial sRNAs are those targeting human host mRNAs. Some sRNAs adopt secondary structures that allow them to interact with human host mRNAs during bacterial infection. Although the function of this class of RNAs has not been fully elucidated yet, their secondary structure could bind not only to specific human mRNAs but also could regulate the expression of individual bacterial genes (Choi et al., 2017). Bacterial sRNAs that mimic eukaryotic miRNAs could target the host immune response during infection. A tRF from *Pseudomonas aeruginosa* downregulates the inflammatory response in both *in vitro* and *in vivo* pulmonary infection models. Indeed, it was shown that this sRNA could downregulate the expression of specific MAPKs involved in the activation of NF-kB, thereby leading to a decreased expression of IL-8 (Koeppen et al., 2016). Recently, Sahr and colleagues reported that the sRNAs RsmY and tRNA-Phe from *Legionella pneumophila* (*L. pneumophila*) could base pair with eukaryotic mRNAs either in the coding region or the untranslated region (UTR) (Sahr et al., 2022). This binding leads to the inhibition of proteins involved in the formation of the RIG-I-like receptor as well as those involved in the Toll-like receptor signalling pathway, such as RIG-I, cRel, and IRAK1 (Sahr et al., 2022).

Furthermore, it has been reported that several bacterial pathogens produce sRNAs that can be secreted within EVs produced by Gram-positive bacteria or outer membrane vesicles (OMVs) produced by Gram-negative bacteria and, by these means, transferred into eukaryotic cells and/or to other bacteria (Ahmadi Badi et al., 2017; Choi et al., 2017; Ahmadi Badi et al., 2020; Stanton, 2021). For instance, *L. pneumophila* releases EVs containing sRNAs both *in vitro* and *in vitro* during infection; these sRNAs modulate the host innate immune response in argonaute-2 (Ago2)-dependent manner (Sahr et al., 2022). Ago2 as a component of the RNA-induced silencing complex (RISC), involved in several cellular processes and functions in RNA-mediated gene silencing (RNAi) (Müller et al., 2020). Taken together, in the host–pathogen interaction scenario, pathogens could actively alter the host machinery for their own benefits by changing the pattern of host mRNA expression (Sarshar et al., 2022a). However, additional researches are necessary to elucidate how bacterial sRNAs delivered by OMVs/EVs mediate inter-kingdom communication upon entering human cells.

### The role of *A. baumannii* sRNAs in different networks of interactions

**FIGURE 1:**
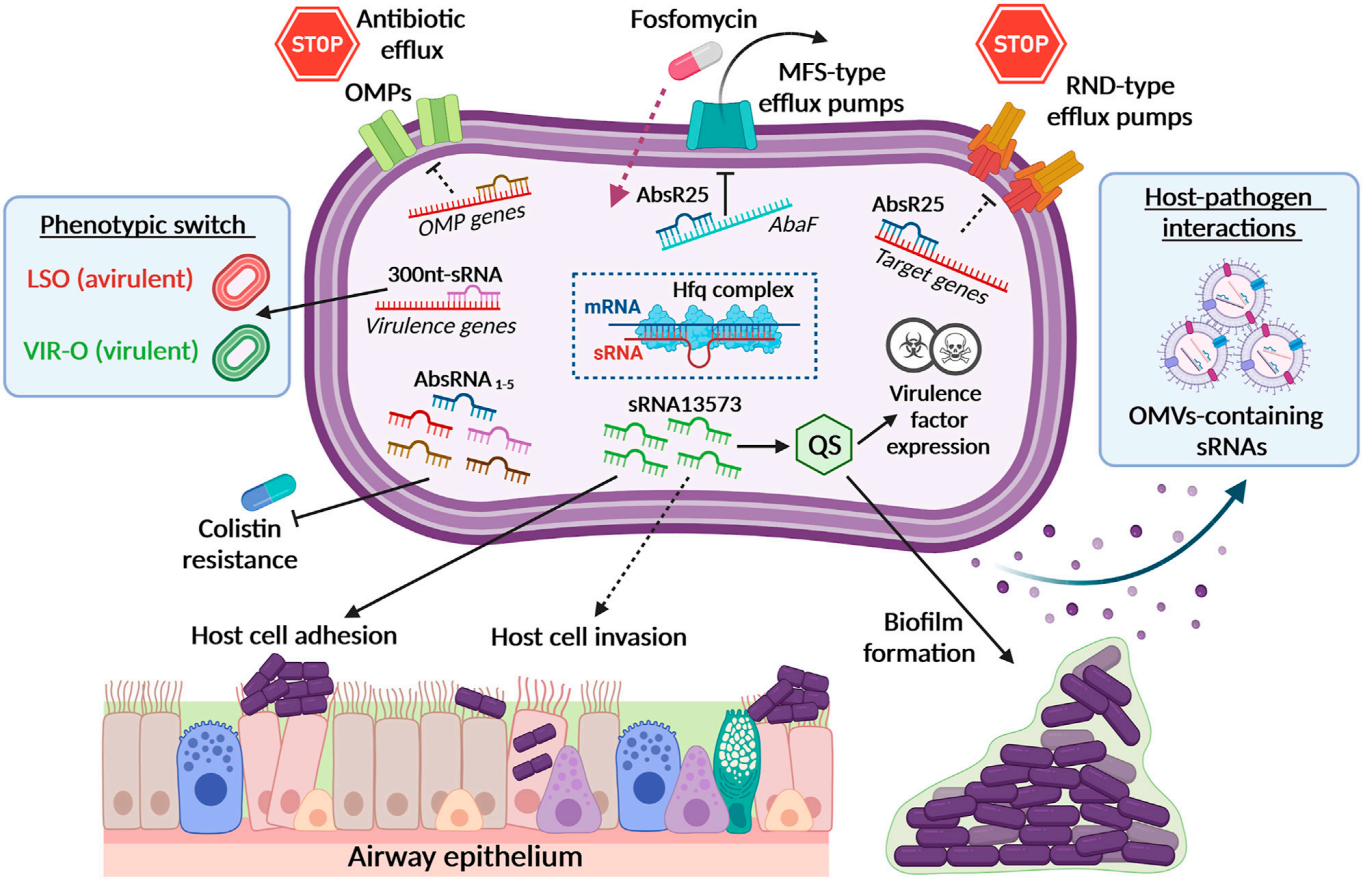
Small RNAs (sRNAs) in *A. baumannii* physiology, antibiotic-resistance and virulence. The figure summarizes *A. baumannii* sRNAs associated with their targets and relative functions. Black dotted lines represent putative or underexplored roles. By the downregulation of the *abaF* gene, sRNA AbsR25 affects the function of the major facilitator superfamily (MFS) transporter, thereby leading to an increased susceptibility to fosfomycin. Other sRNAs can regulate the expression of other efflux pumps and outer membrane proteins (OMPs). The sRNA 13573 was found to be overexpressed during *A. baumannii* biofilm formation as well as during airway epithelial cell adhesion, probably belonging to the Quorum Sensing (QS) Regulatory RNAs. Additional sRNAs are believed to control the expression of *A. baumannii* virulence factors. The identification of differentially regulated sRNAs linked to colistin resistance in COLR *A. baumannii* strains provides the basis for their use as diagnostic markers. A 300 nt-long sRNA has been found to be overexpressed in a subpopulation of opaque virulent colonies showing low rate of translucent switch, named low-switching opaque variants (LSO). Additionally, *A. baumannii* can release sRNAs-containing outer membrane vesicles (OMVs) that may have a role in host-pathogen interactions. *A. baumannii* Hfq complex (i.e., Hfq, mRNA and sRNA) depicted in the centered dotted square represents the main regulatory machinery able to modulate the expression of several fundamental proteins, such as OMPs or to impair sRNA stability and influence bacterial pathogenicity. All the items depicted in this picture have been fully described in the main text.

Hospital-acquired infections caused by multidrug resistant (MDR) bacteria remain an unresolved problem in healthcare settings worldwide. Recently, *A. baumannii* emerged as one of the major opportunistic nosocomial and community-acquired pathogens; its propensity to acquire multidrug, extensive drug and even pandrug resistance phenotypes reduced effective therapies and increased mortality rates (Dehbanipour and Ghalavand, 2022). Apart from pneumonia, several others infections such as wound infections, meningitis, endocarditis, osteomyelitis, endophthalmitis and urinary tract infections (UTIs) have been reported in adults as well as children (Hu & Robinson, 2010; Di Venanzio et al., 2019; Sarshar et al., 2021). The *A. baumannii* ability to adhere and form biofilms on both biotic and abiotic surfaces is the cause of several biofilm-mediated infections such as catheter-associated UTIs (CAUTIs) in clinical settings (Di Venanzio et al., 2019). Within the last decade, several studies tried to unravel the complex mechanisms that led to the emergence of *A. baumannii* as a formidable human pathogen, from its pathogenicity to resistance strategies (Ambrosi et al., 2017; Scribano et al., 2019; Ambrosi et al., 2020; Pompilio et al., 2021; Marazzato et al., 2022). However, these researches have been hampered by the wide heterogeneity of *A. baumannii* isolates owing to its high genome plasticity (i.e., capability to acquire extracellular DNA, high frequency of homologous recombination and elevated presence of mobile elements), that widens its ability to adapt and persist in hospital settings where the presence of these kind of pathogens is constantly monitored (Gaiarsa et al., 2019; Leal et al., 2020; Whiteway et al., 2022). Gene expression modulation by transcription factors (TFs), two-component systems (TCSs), *σ* factors and sRNA-mediated mechanisms are among the preferred bacterial strategies to fight environmental stresses (i.e., oxidation, acidic environment, osmotic or temperature gradients, nutrient starvation, and antibiotic exposure) (Felden & Cattoir, 2018; Allen et al., 2020). In particular, it was recently shown that bacterial trans-encoded sRNAs could modulate antibiotic resistance mechanisms through several strategies, such as by acting on efflux pumps, membrane transporters, LPS and/or cell wall biosynthetic modifications, DNA mutagenesis and biofilm synthesis, thereby fine tuning the responsiveness to a broad spectrum of antibiotics. Detailed reviews on this topic have appeared in the last few years (Dersch et al., 2017; Mediati et al., 2021). However, the overall properties of trans-encoded sRNAs in *A. baumannii* remain elusive compared to other Gram-negative species. Of note, carbapenem-resistant *A. baumannii* is a major concern owing to the enhanced expression of carbapenamases, several classes of efflux pumps and outer membrane proteins (OMPs), which have been reported to be correlated with the capability of this pathogen to form biofilm (Pompilio et al., 2021; Sarshar et al., 2021). Conversely, the sRNAs regulating efflux pumps (EPs) have been extensively studied in *E. coli*, *Clostridium acetobutylicum*, *Mycobacterium tuberculosis*, *Shigella sonnei* and *S. enterica* ser. Typhimurium (Yu & Schneiders, 2012; Chan et al., 2017; Felden & Cattoir, 2018; Gan & Tan, 2019). For example, in *Enterobacteriaceae*, the homeostasis of major OMPs, such as OmpA, OmpC, OmpD, and OmpF, is controlled by the regulatory action of several sRNAs (Guillier et al., 2006; Papenfort et al., 2006; Klein & Raina, 2017; Matera et al., 2022). As these sRNAs have a pivotal role as virulence factors and in drug resistance of several OMPs in *A. baumannii*, mainly OmpA, BamA, LptD, Omp33–36, OmpW, CarO, and OprD, it would be interesting to investigate whether specific sRNAs could either suppress or enhance the expression of these OMPs in *A. baumannii* (Figure 1, top left).

Pathania and colleagues were the first group who investigated the presence of regulatory sRNAs and their functionality in *A. baumannii*. They identified distinct sets of novel differentially expressed sRNAs in *A. baumannii* strain MTCC 1425 compared to ATCC 17978, suggesting that in *A. baumannii* the expression of sRNAs is strain-specific (Sharma et al., 2014). In this screening, the authors found a sRNA, designated AbsR25. By target gene prediction, AbsR25 was initially recognized as the regulator of an efflux pump in *A. baumannii*, therefore suggesting that it might be either directly or indirectly involved in the expression of a transporter and potentially also in drug resistance mechanisms (Sharma et al., 2014) (Figure 1, top right). Later on, Sharma and colleagues demonstrated that the sRNA AbsR25 negatively regulates the expression of the *abaF* gene, which belongs to the major facilitator superfamily (MFS) transporter. AbaF actively effluxes fosfomycin, rendering the cells resistant. Accordingly, its expression is upregulated upon exposure to this antibiotic (Figure 1, top center). Inhibition of *abaF* by AbsR25 lowered fosfomycin minimum inhibitory concentration (MIC) by eightfold but also decreased biofilm formation and virulence in *A. baumannii* (Sharma et al., 2016). Additionally, Alvarez-Fraga and colleagues analyzed the regulatory effects of *A. baumannii* sRNAs on biofilm formation both *in vitro* and *in vivo* (Alvarez-Fraga et al., 2017). Among the differentially expressed sRNAs, the expression level of the sRNA 13573 was significantly higher in biofilm as well as during adhesion to A549 human alveolar epithelial cells, compared to planktonic *A. baumannii* ATCC 17978 cells (Figure 1, middle center and bottom center) (Alvarez-Fraga et al., 2017). Although the mechanism(s) underlying this regulation remains to be fully determined, the involvement of sRNAs in the adhesion to epithelial cells highlights the unrealized key role of sRNAs in *A. baumannii*-host interaction to establish a successful infection.

So far, the comprehensive transcriptomic analysis of *Acinetobacter* spp. led to the discovery of 110 potential sRNAs, the majority of which are conserved in *A. baumannii* and, to a lesser extent, in *Acinetobacter nosocomialis* and *Acinetobacter pittii* (Kröger et al., 2018). Kröger’s group improved the resolution of sRNA *de novo* discovery by differential RNA-seq pooled from sixteen different growth conditions in *A. baumannii* ATCC 17978. Their results indicated that the majority of sRNAs were located within intergenic regions of the bacterial genome or antisense with respect to coding regions or within the 3′ regions of coding genes. Interestingly, 22 sRNAs were found within this latter location and all possessed their own promoters located upstream of the coding gene (Kröger et al., 2018). However, the authors did not explore the possible targets of these 22 sRNAs.

Recently, Cafiso et al. identified several msRNAs that are differentially expressed in colistin resistant (COL R) *A. baumannii* strains through both experimental and computational analyses (Figure 1, bottom left). Although identified as precursors of their mature microRNA-size small RNA form (pre-microAbsRNA), computational prediction of their targets revealed their involvement in the regulation of different biological functions (i.e., biofilm production, virulence and aminoglycoside-resistance), highlighting their role in host-microbe interactions (Cafiso et al., 2020). The innovative aspect of this work is the identification of downregulated sRNAs in COLR vs. colistin susceptible *A. baumannii* strains that could be used to rapidly identify resistant isolates. Most importantly, despite several sRNAs have been identified in the MDR *A. baumannii* strain AB5075, their physiological function(s) remain uncertain so far. Weiss and colleagues identified 78 novel short and conserved strain-specific sRNAs that are present in large copy numbers within the AB5075 genome, using a RNA-seq-based approach (Weiss et al., 2016). Two more sRNAs, Aar and AbsR28 were previously reported in *Acinetobacter baylyi* and *A. baumannii* ATCC 17978, respectively (Schilling et al., 2010; Sharma et al., 2014). Among the others and based on internal homology, they identified six groups of sRNAs with one sRNA particularly abundant and homologous to regulatory C4 antisense RNAs found in bacteriophages P1 and P7. Four additional highly conserved RNA species were identified in strain AB5075, including the signal recognition particle (SRP) RNA, 6S RNA, tmRNA and RNase P RNA, annotated as ABUWs030, ABUWs053, ABUWs059, and ABUWs062. Interestingly, three of them, ABUWs030, ABUWs053, and ABUWs059, were among the most highly expressed ncRNAs in *A. baumannii*, suggesting their potential physiological relevance in this pathogen (Weiss et al., 2016).

Recently, the expression of a ∼300-nt sRNA has been found to be involved in controlling the phenotypic switch from the virulent opaque (VIR-O) to the avirulent translucent (AV-T) phenotype in strain AB5075 (Anderson et al., 2020). This sRNA was encoded at the 5′ end of the *aadB* gene within resistance island two and showed a variable expression. In fact, gene expression analysis revealed that bacterial opacity switching rate varied from VIR-O to the low-switching opaque variants (LSO) as a function of the copy number of this locus (Figure 1, middle left). The LSO represents a subpopulation of cells that exhibit dramatically reduced levels of switching to AV-T relative to that for the VIR-O. This identified sRNA affected virulence, as the LSO cells exhibited decreased virulence *in vivo*. In addition, more than 100 genes were identified as differentially expressed between VIR-O and LSO, suggesting that the ∼300-nt sRNA may act as a global regulator (Anderson et al., 2020).

As already outlined, Hfq can regulate the interactions between mRNA and sRNA, both positively and negatively. Among RNA chaperones, Hfq has been the only one functionally studied in *Acinetobacter* spp. (Figure 1, middle center). Indeed, recent analysis of Hfq in *A. baylyi* and *A. baumannii* ATCC 17978 revealed that deletion of *hfq* in *A. baumannii* impaired growth with respect to the wild-type strain, increased susceptibility to environmental stressors, including desiccation, decreased carbon metabolism and reduced host cell adhesion and virulence (Kuo et al., 2017). The same pleotropic effects observed in *A. baylyi* and *A. baumannii* ATCC 17978 were also shown in *Salmonella* and *E. coli* *hfq* mutants, including reduced growth rates, elevated sensitivity to environmental stresses, defeated OMV production, fimbriae, biofilm formation and adhesion, invasion and survival in eukaryotic cells (Sittka et al., 2008; Hayashi-Nishino et al., 2012; Kuo et al., 2017). While it appears that Hfq is a vital virulence factor in *A. baumannii*, it remains unclear how this chaperone is involved in mediating sRNA-mRNA interactions, a function that is worth to be explored.

### Conclusion and future perspectives

Host-pathogen interactions are based on the production of specific types of communication molecules. Among them, non-coding small RNAs, sRNAs, received an increased attention as they are directly involved in the regulation of gene expression, resulting in a rapid phenotypic change and fast adaptation to the environment. The study of eukaryotic miRNAs started about 30 years ago and a huge knowledge on their targets and functions are nowadays available. Owing to this, several miRNAs are currently used as disease biomarkers, they are targets for new therapeutics (anti-miR compounds) or under therapeutic evaluation in several clinical trials. Vice versa, the massive discovery of bacterial sRNAs, the similarity with human miRNAs, and detailed functional studies are very recent concepts and the role of bacteria sRNAs in host-pathogen interaction is still an unexplored field. Only few studies reported that secreted bacterial RNAs can be stably transported either in OMVs or as complexes with proteins/biomolecules (Ghosal, 2018; Quendera, et al., 2020) or found in free-form circulating in the human plasma of healthy donors (Wang and Fu, 2019). sRNA-dependent gene regulation influences bacterial lifestyle, modulating the switch from a “harmless” colonizer to a pathogen. However, the study of the expression profiles of bacterial sRNAs has not been investigated in details yet, neither in healthy individuals nor in infected patients. We think that further studies about bacterial sRNAs could help to better understand the role of these molecules as a diverse toolkit for bacterial adaptation to the host environment (Sarshar et al., 2017; Ambrosi et al., 2019; Sarshar et al., 2022b) or as potentially novel therapeutics (i.e., administration of natural or synthetic oligonucleotides) (Markelova et al., 2021; Ozoline and Shavkunov, 2021). Being an opportunistic pathogen, *A. baumannii* represents an excellent model to characterize bacterial sRNAs and evaluate their role in *A. baumannii* antibiotic-resistance and pathogenesis. This is particularly true for those sRNAs packed into OMVs that can be directly absorbed by the host cells (Figure 1, middle right). However, the studies on the functional role of bacterial secreted RNAs in infected host cells has only recently started to appear and achieved results are insufficient for depicting the whole crosstalk during bacterial infection. Indeed, the majority of the studies were conducted using *in vitro* or animal models that do not consider the human tissue architecture and function. Hence, application of human-derived advanced cell cultures, such as organoids, together with high resolution RNA-seq techniques will enable us to understand the impact of sRNAs on infectious diseases. These models will allow the molecular characterization of the interactions between bacterial RNAs and host factors and will clarify their roles and importance in bacterial pathogenicity, thereby including them under the general concept of “RNA effector.” Last but not least, owing to the presence of specific sRNAs in MDR bacteria and/or their differential expression upon antibiotic stress, this field of research opens the possibility to develop alternative and innovative therapeutic strategies based on sRNAs.
